# Central Obesity and Albuminuria: Both Cross-Sectional and Longitudinal Studies in Chinese

**DOI:** 10.1371/journal.pone.0047960

**Published:** 2012-12-12

**Authors:** Wen-Yuan Lin, F. Xavier Pi-Sunyer, Chiu-Shong Liu, Chia-Ing Li, Lance E. Davidson, Tsai-Chung Li, Cheng-Chieh Lin

**Affiliations:** 1 Department of Family Medicine, China Medical University Hospital, Taichung, Taiwan; 2 Medical Research, China Medical University Hospital, Taichung, Taiwan; 3 School of Medicine, China Medical University, Taichung, Taiwan; 4 Graduate Institute of Clinical Medical Science, China Medical University, Taichung, Taiwan; 5 Graduate Institute of Biostatistics, China Medical University, Taichung, Taiwan; 6 Institute of Health Care Administration, College of Health Science, Asia University, Taichung, Taiwan; 7 New York Obesity and Nutrition Research Center, St. Luke's-Roosevelt Hospital, Columbia University–College of Physicians and Surgeons, New York, New York, United States of America; 8 Department of Internal Medicine, University of Utah, Salt Lake City, Utah, United States of America; University of Louisville, United States of America

## Abstract

**Background:**

Albuminuria is recognized as a marker of vascular dysfunction. Central obesity increases the risk of cardiovascular disease. Little is known about the association between albuminuria and central obesity in Chinese. We aimed to assess the association between central obesity and prevalence and incidence of albuminuria in a middle-aged population-based cohort study.

**Methods:**

This is a cross-sectional and longitudinal cohort study. A total of 2350 subjects aged ≥40 years were recruited in 2004 in Taiwan for cross-sectional analysis. Longitudinal analysis included 1432 baseline normoalbuminuria subjects with a mean 2.8 years follow-up, 67 of whom exhibited incident albuminuria. Albuminuria was defined as urinary albumin-to-creatinine ratio ≥30 mg/g creatinine. Multiple logistic regression analyses were used to evaluate the relationship between central obesity and prevalence and incidence of albuminuria after adjustment for age, gender, body mass index, blood pressure, renal function, glucose, high sensitivity c-reactive protein, smoking, betel nut chewing, alcohol drinking, and physical activity.

**Results:**

At baseline, albuminuria is significantly associated with central obesity. The adjusted odds ratio of having albuminuria among subjects with central obesity was 1.73(95% confidence interval (CI): 1.04–2.85), compared to the subjects without central obesity. In multivariable models, participants with central obesity at baseline had a 112% increase in risk of incident albuminuria (adjusted incidence rate ratio (95% CI): 2.12(1.01–4.44)) compared with participants with non-central obesity.

**Conclusions:**

Abdominal adiposity was independently associated with increased prevalence and incidence of albuminuria in Chinese. The mechanisms linking adiposity and albuminuria need to be addressed.

## Introduction

End-stage renal disease (ESRD) is an important and burdensome disease worldwide. Data from the US Renal Data System (2011) reported that Taiwan is the country with the highest incidence and prevalence of ESRD [1]. Albuminuria is an early marker of vascular dysfunction and renal disease which has been linked to an increased risk for future cardiovascular disease, ESRD, and cardiovascular disease (CVD)/all-cause mortality [2], [3], [4], [5]. Albuminuria is not only common in people with specific diseases, such as diabetes or hypertension but also in the general population [6]. To identify the early risk factors of albuminuria had become very important in Taiwan as well as other countries with a high prevalence of ESRD.

Obesity has been recognized as a serious health problem, leading to an increased risk of many chronic diseases, such as hypertension, type 2 diabetes, lung function impairment, chronic kidney disease, cardiovascular disease, and cancer [7], [8], [9], [10], [11] and associated with increased CVD and all-cause mortality [12], [13]. The prevalence of obesity has increased dramatically globally [14]. The World Health Organization (WHO) has estimated that approximately 1.6 billion are overweight and at least 400 million adults are obese [15]. It further estimates that approximately 2.3 billion adults will be overweight and more than 700 million will be obese by 2015 [15]. In the United States, the prevalence of obesity in adults doubled between 1986 and 2000 and it is anticipated that 3 of every 4 adults will be overweight or obese by the year 2020 [16], [17]. While the prevalence of obesity is high and rising in developed countries, the increase is often faster in developing nations. For example, in the Nutrition and Health Surveys in Taiwan of 1993–1996 and 2005–2008, the prevalence of overweight or obesity (body mass index (BMI) ≥24 kg/m^2^) increased from 33.4% to 50.8% in adult men and from 19.7% to 24.8% in adult women, and central obesity (waist circumference ≥90 cm in men and/or ≥80 cm in women) increased from 31.7% to 36.9% and 12.2% to 33.6% in men and women, respectively [18]. Obesity consists of excessive fat deposits throughout in the body, whereas central obesity denotes excessive fat in the mid-body region, much of it in the intra-abdominal area. Compared with overall obesity, central obesity appears to be more strongly associated with CVD risk factors and other chronic diseases [12], [19], [20], [21]. Only a few studies have reported that albuminuria is associated with central obesity [22], [23]. For example, Bonnet et al. reported that elevated waist circumference is related to the development of elevated albuminuria in non-diabetic subjects [23]. These studies, however, either had small sample sizes, were cross-sectional studies, were done on Caucasian, or were focused on subjects with a specific condition, such as non-diabetic subjects. We conducted a population-based cohort study in a metropolitan city in Taiwan to investigate the association between central obesity and prevalence and incidence of albuminuria in Chinese middle-aged adults.

## Materials and Methods

### Baseline subjects

The target population consisted of residents of Taichung city, Taiwan who were age 40 and above in October, 2004, as described in previous reports [2], [24]. There were a total of 363,543 residents in this area during the time of study, which represented about 4.09% of the national population of the same age. A Two-stage sampling design was used to draw residents, with sampling rate proportional to size within each stage. 4280 individuals were randomly selected. During household visits, we identified 750 individuals that were ineligible and excluded them from the study sample. The reasons for exclusion included death (n = 18), hospitalization or imprisonment (n = 14), living abroad (n = 39), moving out (n = 411), living in their children's house (n = 7), mistake of the sampling frame (n = 59), and not being at home during 3 visits made by interviewers (n = 202). Among 3530 individuals selected, 2359 agreed to participate. Subjects with incomplete data for urine albumin test were excluded. The final sample was 2350 subjects. The selected subjects (n = 2350) and deleted subjects (n = 9) were not statistically significantly different in age, height, weight, BMI, waist circumference, and hip circumference.

### Follow-up cohort subjects

**Figure 1:**
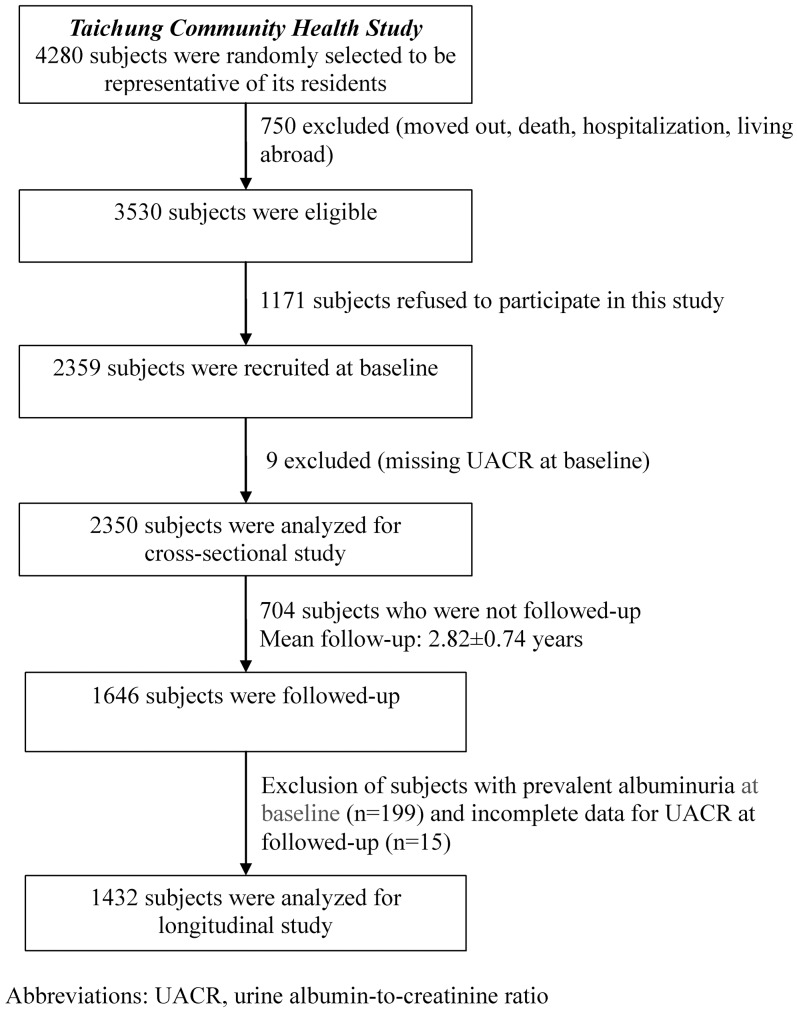
Taichung Community Health Study (TCHS) population selection.

After baseline measurement, subjects were invited to participate in the follow-up study. The study population selection is depicted in **Figure 1**. Among these 2350 subjects, 1646 subjects (overall follow-up rate, 71.3%) agreed to participate in the follow-up study before the end of July, 2009. Subjects with albuminuria at baseline (n = 199) or that had incomplete data for urinary albumin-to-creatinine ratio (UACR) at follow-up (n = 15) were excluded from further longitudinal analysis. 1432 subjects with a mean 2.8 years follow-up were finally included in the longitudinal analysis, with 67 cases of incident albuminuria.

### Anthropometric index and laboratory assays

Trained staff measured height, waist and hip circumference, weight, and blood pressure (BP) as previously reported [2], [24], [25]. BMI was calculated as weight divided by height squared (kg/m^2^). Central obesity was defined as waist circumference ≥90 cm in men and/or ≥80 cm in women, thresholds that have been developed specific to Asian populations based on the observation that obesity-related complications in Asians occur at lower BMI cut-points than those used in Caucasians [26]. Blood was drawn in the morning after a 12-hour overnight fast and was sent for analysis within 4 hours of collection. High sensitivity C-reactive protein (hsCRP) levels were measured by nephelometry, a latex particle-enhanced immunoassay (TBA-200FR, Tokyo, Japan). The inter-assay and intra-assay CVs were <2.0% and <1.9%, respectively. Diabetes was defines as: (1) fasting plasma glucose concentration ≥7.0 mmol/L (126 mg/dL) and/or (2) diabetes history on oral hypoglycemic agents or insulin treatment.

### Sociodemographic factors and life style behaviors

Age, gender, employment, education, diet habit, and physical activity were collected by self-administered questionnaires. Smoking, alcohol drinking, and betel nut chewing history were divided into 3 classes as follows: never, former, and current. Physical activity status was divided into 2 classes: never/seldom and current.

### Albuminuria and renal function

Renal function was evaluated by estimated glomerular filtration rate (eGFR), which was estimated using the Modification of Diet in Renal Disease formula as follows: eGFR (mL/min/1.73 m^2^) = 186×(serum creatinine (mg/dL))^−1.154^×(age)^−0.203^×(0.742 if female). A UACR≥30 mg/g defined albuminuria and normoalbuminuria was <30 mg/g. Within albuminuria, UACR ranging from 30 mg/g to 300 mg/g was defined as microalbuminuria and above 300 mg/g was macroalbuminuria.

### Statistical analysis

The data are presented as means and standard deviation unless indicated otherwise. Student's t-test was used to compare mean values. Variables with significant deviation from normal distribution were log transformed and assessed by a Kolmogorov–Smirnov test before further analyses. The Pearson's χ^2^ test was used to compare the differences in the categorical variables (such as smoking, alcohol drinking, betel nut chewing, and physical activity) across central obesity or albuminuria status. Multiple logistic regression analyses were used to evaluate the associations between prevalence and incidence of albuminuria and central obesity after adjustment for potential confounders. All statistical tests were 2-sided at the 0.05 significance level. Data were analyzed for the whole cohort and separately for men and women using the PC version of SPSS statistical software (17th version, SPSS Inc., Chicago, IL, USA).

Ethics approval for patient recruitment and data analyses was obtained from the Institutional Review Board of the China Medical University Hospital. All subjects signed an informed consent form before data collection.

## Results

### Albuminuria prevalence: cross-sectional analysis

The cohort included in this analysis comprised 2350 subjects at baseline. **Table 1** shows the baseline characteristics according to central obesity status. Subjects with central obesity were older and had greater height, weight, BMI, waist circumference, systolic and diastolic BP, fasting glucose, hsCRP, UACR, prevalence of diabetes, and lower eGFR than subjects without central obesity. The prevalence of diabetes in baseline cohort was 12.1% (14.1% in men and 10.2% in women). **Table 2** shows the baseline characteristics according to albuminuria level. The overall prevalence of albuminuria was 13.0% (14.1% in men and 11.9% in women). Subjects with albuminuria were older and had greater height, BMI, waist circumference, systolic and diastolic BP, fasting glucose, hsCRP, UACR, prevalence of diabetes, and lower eGFR than subjects without albuminuria. **Table 3** shows unadjusted and adjusted odds ratios (ORs) (95% confidence intervals, CI) of having albuminuria derived from a multiple logistic regression analysis using central obesity as independent variables after adjustment for potential confounders. After adjusting for age, gender, BMI, systolic and diastolic BP, fasting glucose, hsCRP, eGFR, smoking, alcohol drinking, betel nut chewing, and physical activity status, multiple logistic regression analyses revealed that central obesity was significantly associated with albuminuria(p<0.05). Compared to the subjects without central obesity, the adjusted ORs of having albuminuria among subjects with central obesity was 1.73(95% CI: 1.04–2.85) (1.39 (0.69–2.84) in men and 2.35(1.10–5.01) in women) (**Table 3** model 2). In the baseline sample, 39 subjects (19 men, 20 women) had macroalbuminuria. Results from **Table 3** model 2 remained essentially unchanged when these subjects were excluded (the adjusted ORs = 1.66(1.00–2.75), p<0.05).

**Table 1:** Baseline characteristics according to central obesity status (n = 2350)‡.

	With central obesity (n = 787)‡	Without central obesity (n = 1563)‡	*p* value
Age (years)* ^,^ #	59.2±11.7	55.6±11.3	<0.001
Men (n, %)†	384 (48.8%)	758 (48.5%)	0.892
Height (cm)* ^,^ #	161.3±8.5	160.4±7.7	0.029
Weight (kg)* ^,^ #	71.3±10.9	58.9±8.5	<0.001
BMI (kg/m^2^)* ^,^ #	27.3±3.0	22.8±2.3	<0.001
WC (cm)* ^,^ #	91.0±7.6	76.5±7.3	<0.001
Systolic BP (mmHg)* ^,^ #	145.0±21.9	130.9±20.5	<0.001
Diastolic BP (mmHg)* ^,^ #	83.5±12.0	76.6±12.0	<0.001
Fasting glucose(mmol/L)* ^,^ #	6.10±1.83	5.56±1.40	<0.001
UACR (mg/g creatinine)* ^,^ #	37.4±133.0	26.6±165.3	<0.001
hsCRP (mg/L)* ^,^ # ^,^ **	0.29±0.58	0.23±0.49	<0.001
eGFR (mL/min/1.73 m^2^)* ^,^ #	82.5±20.0	87.5±23.6	<0.001
Diabetes (n, %)†	148 (18.8%)	136 (8.7%)	<0.001
Smoking†			0.070
Current	16.5%	15.4%	
Former	13.7%	10.8%	
Never	69.7%	73.7%	
Alcohol drinking†			0.127
Current	21.6%	23.8%	
Former	6.4%	4.6%	
Never	72.0%	71.6%	
Betel nut chewing†			0.186
Current	3.7%	3.5%	
Former	6.7%	4.9%	
Never	89.6%	91.6%	
Physical activity†			
Current	66.5%	67.7%	0.540
Never/seldom	33.5%	32.3%	

Abbreviations: BMI, body mass index; WC, waist circumference; BP, blood pressure; UACR, urinary albumin-to-creatinine ratio; eGFR: estimated glomerular filtration rate; hsCRP, high sensitivity C-reactive protein.

‡ :Central obesity is defined as waist circumference ≥90 cm in men, and/or ≥80 cm in women.

* :Student's *t*-test for unpaired data was used for the comparison of mean values between genders; data are means ± SD;

# :Statistics were tested using the log-transformed values.

† :Pearson's χ^2^ test was used for categorical data; data were shown as percentage.

** :N = 1671.

**Table 2:** Baseline characteristics according to albuminuria level (n = 2350)‡.

	Normoalbuminuria (n = 2045)	Albuminuria (n = 305)	*p* value
Age (years)* ^,^ #	55.9±11.2	63.3±12.0	<0.001
Men (n, %)†	981 (48.0%)	161 (52.8%)	0.116
Height (cm)* ^,^ #	160.9±7.9	159.7±8.8	0.020
Weight (kg)* ^,^ #	62.9±10.8	64.0±12.2	0.164
BMI (kg/m^2^)* ^,^ #	24.2±3.3	25.0±3.8	<0.001
WC (cm)* ^,^ #	80.9±9.9	84.9±10.3	<0.001
Systolic BP (mmHg)* ^,^ #	133.1±20.4	152.6±24.6	<0.001
Diastolic BP (mmHg)* ^,^ #	77.9±11.9	85.5±13.8	<0.001
Fasting glucose (mmol/L)* ^,^ #	5.58±1.26	6.80±2.70	<0.001
UACR (mg/g creatinine)* ^,^ #	6.62±6.27	188.3±396.5	<0.001
hsCRP (mg/L)* ^,^ # ^,^ **	0.22±0.39	0.45±1.04	<0.001
eGFR (mL/min/1.73 m^2^)* ^,^ #	87.3±21.7	76.0±25.7	<0.001
Diabetes (n, %)†	187 (9.1%)	97 (31.8%)	<0.001
Smoking†			0.009
Current	15.5%	17.8%	
Former	11.1%	16.4%	
Never	73.4%	65.8%	
Alcohol drinking†			0.234
Current	23.2%	22.3%	
Former	4.9%	7.2%	
Never	71.9%	70.5%	
Betel nut chewing†			0.603
Current	3.6%	3.0%	
Former	5.4%	6.6%	
Never	91.0%	90.5%	
Physical activity†			
Current	67.4%	66.9%	0.871
Never/seldom	32.6%	33.1%	

Abbreviations: BMI, body mass index; WC, waist circumference; BP, blood pressure; UACR, urinary albumin-to-creatinine ratio; eGFR: estimated glomerular filtration rate; hsCRP, high sensitivity C-reactive protein.

* :Student's *t*-test for unpaired data was used for the comparison of mean values between genders; data are means ± SD;

# :Statistics were tested using the log-transformed values.

† :Pearson's χ^2^ test was used for categorical data; data were shown as percentage.

‡ :Normoalbuminuria: UACR<30 mg/g creatinine; Albuminuria: UACR≥30 mg/g creatinine.

** :N = 1671.

**Table 3:** Unadjusted and adjusted odds ratios (95% confidence intervals) of having albuminuria derived from a multiple logistic regression analysis using central obesity as independent variables in the baseline cohort, adjusted for potential confounders†.

	Model 1*	Model 2**
Without central obesity (overall, n = 1563)‡	1.00(Reference)	1.00(Reference)
With central obesity (overall, n = 787)‡	2.14(1.68–2.73)#	1.73(1.04–2.85)§
Without central obesity (men, n = 758)‡	1.00(Reference)	1.00(Reference)
With central obesity (men, n = 384)‡	1.84(1.31–2.58)#	1.39 (0.69–2.84)
Without central obesity (women, n = 805)‡	1.00(Reference)	1.00(Reference)
With central obesity (women, n = 403)‡	2.52(1.77–3.58)#	2.35(1.10–5.01)§

Abbreviations: BMI, body mass index; BP, blood pressure; eGFR: estimated glomerular filtration rate; UACR, urinary albumin-to-creatinine ratio; hsCRP, high sensitivity C-reactive protein.

* :Model 1: unadjusted.

** :Model 2: adjusted for age, gender, BMI, systolic BP, diastolic BP, hsCRP, fasting glucose, eGFR, smoking, alcohol drinking, betel nut chewing, and physical activity.

† :Normoalbuminuria: UACR<30 mg/g creatinine; Albuminuria: UACR≥30 mg/g creatinine; Reference group is normoalbuminuria.

‡ :Central obesity is defined as waist circumference ≥90 cm in men, and/or ≥80 cm in women.

§ : *p*<0.05.

∥ : *p*<0.01.

# : *p*<0.001.

### Albuminuria incidence: longitudinal analysis

Of 1432 men and women, mean (±SD) age of 58.1±10.3 years with 2.8 mean years of follow-up, 67 (4.7%) developed albuminuria. The incidence rate was 16.6/1000 person-year (non-central obesity group: 11.0/1000 person-years; central obesity group: 31.2/1000person-years). **Table 4** shows the age adjusted incidence rate ratio (IRR) and multivariate adjusted IRR for BMI and central obesity in relation to albuminuria. Participants with central obesity had a 112% increase in the risk of albuminuria (IRR: 2.12, 95% CI:1.01,4.44) compared to participants without central obesity after adjustment for age, gender, BMI, smoking, alcohol drinking, betel nut chewing, physical activity, systolic BP, diastolic BP, hsCRP, fasting glucose, and eGFR. Further stratified by gender, the IRR (95% CI) in men and women was 1.67(0.59–4.76) and 3.13(1.06–9.22), respectively (**Table 4**).

**Table 4:** Incidence rate ratio (95% confidence interval) of incident albuminuria according to body mass index and central obesity in the follow-up cohort†.

	Age-adjusted IRR (95%CI)*	Multivariate IRR (95%CI)**
Overall (n = 1432)		
BMI (kg/m^2^)	1.13(1.05–1.21)#	1.06(0.95–1.19)
Central obesity‡ ^, ^ ***	2.50(1.52–4.13)#	2.12(1.01–4.44)§
Men (n = 707)		
BMI (kg/m^2^)	1.18(1.07–1.30)∥	1.15(0.99–1.34)
Central obesity‡ ^, ^ ***	2.73(1.37–5.43)∥	1.67(0.59–4.76)
Women (n = 725)		
BMI (kg/m^2^)	1.10(1.00–1.22)	0.98(0.82–1.16)
Central obesity‡ ^, ^ ***	2.55(1.21–5.36)§	3.13(1.06–9.22)§

Abbreviations: IRR, incidence rate ratio; BMI, body mass index; BP, blood pressure; eGFR: estimated glomerular filtration rate; UACR, urinary albumin-to-creatinine ratio; hsCRP, high sensitivity C-reactive protein.

* :Adjusted for age for men and women and adjusted for age and gender for overall cohort.

** :Adjusted for age, gender, BMI, smoking, alcohol drinking, betel nut chewing, physical activity, systolic BP, diastolic BP, hsCRP, fasting glucose, and eGFR.

*** :Without central obesity as reference group.

† :Normoalbuminuria: UACR<30 mg/g creatinine; Albuminuria: UACR≥30 mg/g creatinine; Reference group is normoalbuminuria.

‡ :Central obesity is defined as waist circumference ≥90 cm in men, and/or ≥80 cm in women.

§ : *p*<0.05.

∥ : *p*<0.01.

# : *p*<0.001.

## Discussion

In this study, we demonstrate that central obesity is significantly associated with albuminuria in middle-aged Chinese, even with adjustment for general obesity, hsCRP, renal function and lifestyle factors. We also found that subjects with central obesity tend to develop albuminuria, independently of BMI. These findings were most pronounced in women.

The association between central obesity and albuminuria has been previously reported [22], [27]. For example, in the Look AHEAD (Action for Health in Diabetes) Study, increased BMI and abdominal obesity (measured by waist circumference) were found to be associated with albuminuria in overweight and obese adults with type 2 diabetes [27]. de Boer et al. also found that waist circumference predicts the subsequent development of microalbuminuria in type 1 diabetes [28]. Thoenes et al found that abdominal obesity is associated with microalbuminuria and an elevated cardiovascular risk profile in patients from 26 countries with hypertension [29]. Chandie Shaw et al. also reported that central obesity (measured by waist-to-hip ratio) is independent risk factor for increased albuminuria in normoglycemic South Asian subjects [22]. A similar finding was observed in the Copenhagen City Heart Study [30]. These studies provide an agreement that adiposity is associated with albuminuria. However, these studies are either cross-sectional studies or focused on specific conditions or diseases, such as diabetes or hypertension subjects. Our study is a population-based cohort survey which demonstrates that central obesity is associated with an increased risk of prevalence and incidence of albuminuria in Chinese. In addition, this study provides confirmatory data that the reduced cut-points for abdominal obesity specific to Asian populations are sensitive predictors of albuminuria, similar to thresholds used in Caucasians.

Sex difference is another point worthy of mention in this study. Foster et al. reported that, in the Framingham Heart Study, visceral adipose tissue is associated with microalbuminuria in men but not in women [31]. Our result showed that central obesity is more strongly associated with albuminuria in women than in men. Our previous study also found that hsCRP is closely associated with metabolic syndrome in women than in men [32]. Sullivan et al. reported that the renal protection afforded to female hypertensive rats is associated with lower blood pressure, decreased macrophage infiltration, and decreased levels of oxidative stress [33]. In this gender difference, sex hormones may play an important role on the inflammatory mechanism which links central obesity and albuminuria, and these merit further study.

Several mechanisms link central obesity to albuminuria have been proposed. Firstly, compared to general obesity, central obesity is more strongly associated with inflammatory markers and oxidative stress which may increase the risks of CVD and other chronic diseases, such as hypertension or diabetes [19], [25], [29], [34], [35], [36], [37]. These chronic diseases are closely related to microalbuminuria [25]. In the current study, we also found that subjects with central obesity or albuminuria had higher hsCRP level than non-central obesity or normoalbuminuria. Second, studies reported that the adipocyte hormone adiponectin and leptin, which can regulate podocyte function, may play an important role in the development of obesity-related albuminuria [38], [39], [40]. A possible mechanism by which central obesity promotes glomerular damage and induces microalbuminuria is through decreasing serum adiponectin and increasing serum leptin levels [38], [41]. Third, central obesity is an underlying feature of insulin resistance. Subjects with insulin resistance may increase urinary albumin excretion, leading progressively to chronic kidney disease [42], [43]. Therefore, central obesity may, through insulin resistance, increase the risk of albuminuria and glomerular damage. Fourth, central obesity may activate the transforming growth factor beta (TGF-β)-related mechanisms, leading to glomerular hyperfiltration. Glomerular hyperfiltration causes renin-angiotensin-system (RAS) activation which leads to renal damage [37], [44]. Previous studies reported that the use of angiotensin-converting-enzyme inhibition in patients with uncomplicated type 1 DM results in a significant decline in hyperfiltration [44], [45], [46]. Obesity also increases renal tubular sodium reabsorption which impaired renal pressure natriuresis [46]. Fifth, central obesity may induce hyperleptinemia through sympathetic nervous system activation, thus stimulating the hypothalamic pro-opiomelanocortin pathway to cause renal damage [46], [47].

A strength of this study is that it uses a population-based prospective cohort not limited to specific conditions or diseases, and as such may be more representative of the general population than previous studies. Second, we demonstrate the strong association between central obesity and albuminuria in the baseline population using a cross-sectional design. Our study design enabled us to infer causality of central obesity on albuminuria incidence after 3 years of follow-up. Third, our sample size is large enough to enable adjustment of potential confounders to minimize bias. One limitation associated with this study was that the diagnosis of albuminuria was depended on a spot urine sample, which may not represent the true level of urinary albumin excretion. The potential misclassification, however, is likely non-differential with our results. Second, our findings may not be generalizable to young adults because we recruited subjects aged 40 and older. Third, the purely Chinese sample may limit the generalizability of our results to other races or ethnic populations.

In summary, subjects with central obesity had increased prevalence and incidence of albuminuria in a population-based middle-aged Chinese cohort. This association was stronger in women than in men. Albuminuria may be one of the major complications of central obesity. Obesity-associated renal disease should be prevented or treated by weight reduction.
